# Updated Greek Rheumatology Society Guidelines for the Management of Rheumatoid Arthritis

**DOI:** 10.31138/mjr.31.1.163

**Published:** 2020-06-11

**Authors:** Dimitrios Vassilopoulos, Spiros Aslanidis, Dimitrios Boumpas, George Kitas, Spyridon N. Nikas, Dimos Patrikos, Petros P. Sfikakis, Prodromos Sidiropoulos

**Affiliations:** 1 Joint Rheumatology Program, 2^nd^ Department of Medicine and Laboratory, National and Kapodistrian University of Athens, School of Medicine, Hippokration General Hospital, Athens, Greece; 2 Private Practice, Thessaloniki, Greece; 3 Joint Rheumatology Program, 4^th^ Department of Medicine, National and Kapodistrian University of Athens, School of Medicine, Attikon University General Hospital, Athens, Greece; 4 Hygeia Hospital, Athens, Greece; 5 Private Practice, Ioannina, Greece; 6 Metropolitan Hospital, Piraeus, Greece; 7 Joint Rheumatology Program, 1^st^ Dept of Propaedeutic & Internal Medicine, National and Kapodistrian University of Athens, School of Medicine, Laiko General Hospital, Athens, Greece; 8 Clinical Immunology and Allergy Department, Medical School, University of Crete, Heraklion, Greece

**Keywords:** Rheumatoid arthritis, guidelines, therapy, biologics, disease modifying anti-rheumatic drugs

## INTRODUCTION

The Greek Rheumatology Society and the Greek Association of Professional Rheumatologists (EREEPERE) has been issuing treatment Guidelines for rheumatoid arthritis (RA) since 2005. These Guidelines have been updated in 2009 and 2012.

Here we present the updated Guidelines for the treatment of RA prepared by the *Special Committee of Diagnostic and Therapeutic Protocols in Rheumatic Diseases* of ERE-EPERE and input from experts in the field. In the preparation of these Guidelines the most recent Guidelines from the American College of Rheumatology (ACR)^1^, the Recommendations and Treat To Target paradigm from the European League against Rheumatism (EULAR)^2–5^ were taken into account.

## GENERAL PRINCIPLES OF THERAPY

Rheumatoid arthritis is the most common, chronic, autoimmune inflammatory arthritis in the Greek population that, without timely and effective treatment, leads to permanent joint or extra-articular damage, disability, impaired quality of life and decreased survival.

The following General Principles apply to the treatment of RA in daily clinical practice:

1. RA is managed by the **rheumatologist**, and therapeutic choices are based on a shared decision process between the rheumatologist and the well-informed patient.
2. Treatment of RA should be initiated **immediately after the diagnosis** of the disease for better treatment outcomes and prevention of permanent joint damage.
3. Assessment of disease activity should be made with established **indices of disease activity** such as the Disease Activity Score (DAS) 28 – ESR, (*Supplementary Table 1*).
4. Treatment targets include sustained **remission (DAS28-ESR < 2.6)** or, if this is not possible, **low disease activity (DAS28-ESR < 3.2)** for all RA patients (*Supplementary Table 2*).
5. To achieve the above therapeutic goals, frequent monitoring of patients every **1–3 months** (for those with moderate/high disease activity) or **3–6 months** (for those with low disease activity or in remission).
6. Treatment efficacy is assessed **3–6 months** after treatment initiation or modification.
7. **The criterion for changing or discontinuating treatment** is the inability to achieve low disease activity **(DAS28-ESR > 3.2).**
8. Treatment decisions are based on **disease activity**, **patients’ preferences,** presence or absence of **adverse prognostic factors,** presence of **comorbidities** and the occurrence of **side effects** from the administered therapies.

**Therapeutic steps**

**Figure 1.**
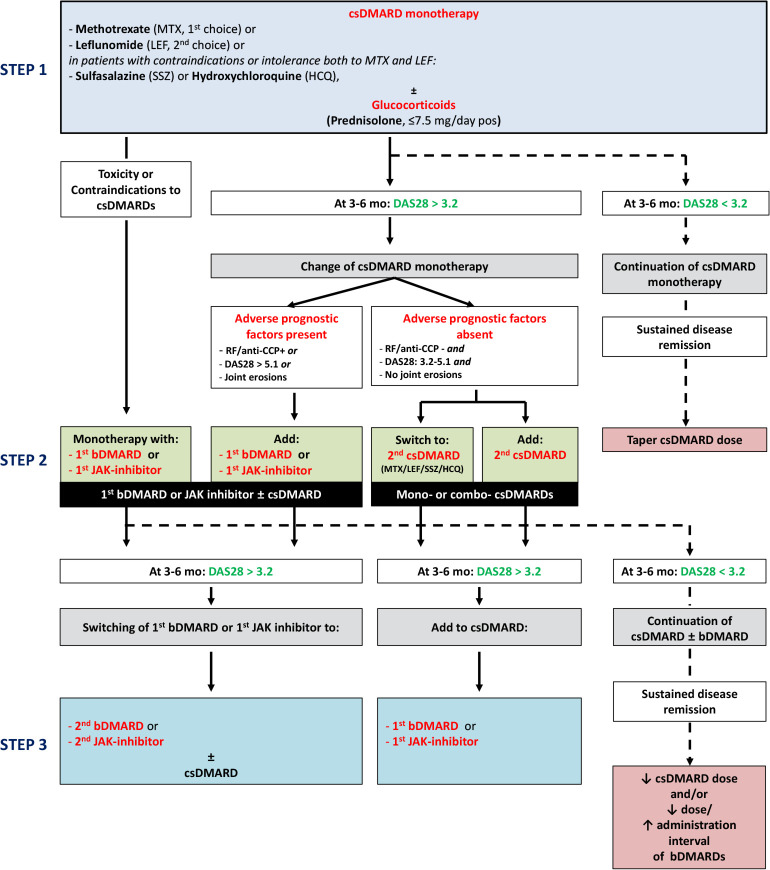
Therapeutic algorithm of rheumatoid arthritis A 3-step algorithm for the management of patients with rheumatoid arthritis is shown. csDMARD: conventional synthetic disease-modifying anti-rheumatic drug, RF: rheumatoid factor, anti-CCP: anti-cyclic citrullinated peptide antibodies, mo: months, DAS28: Disease activity score 28, bDMARD: disease-modifying anti-rheumatic drug, JAK: janus kinase **1st bDMARD** **Anti-TNFs:** Adalimumab, Certolizumab Pegol, Etanercept, Golimumab, Infliximab **Non-anti-TNFs**: Abatacept, interleukin-6/IL-6 inhibitors (Tocilizumab or Sarilumab*), IL-1 inhibitors (Anakinra) or in selected patients (see main text for details): B-cell depleting agents (Rituximab) or **Their approved biosimilar** **JAK inhibitors:** Tofacitinib, Baricitinib (EMA approved), Upadacitinib (EMA approved) **2nd bDMARD** **Anti-TNFs:** Adalimumab, Certolizumab Pegol, Etanercept, Golimumab, Infliximab **Non-anti-TNFs**: Abatacept, interleukin-6/IL-6 inhibitors (Tocilizumab or Sarilumab*), IL-1 inhibitors (Anakinra) or B-cell depleting agents (Rituximab) or **Their approved biosimilar** **JAK inhibitors:** Tofacitinib, Baricitinib (EMA approved), Upadacitinib (EMA approved)

The recommended 3 steps in the treatment of RA are shown in *Figure 1*. Most specifically:

*Step 1*
  1. The **initial treatment step is** the administration of conventional synthetic disease-modifying anti-rheumatic drugs **(csDMARDs)** as **monotherapy**:
    - The 1^st^ option is **methotrexate** (**MTX)** at a dose of 15–25 mg/week pos or subcutaneously in combination with folic acid (5 mg/week pos).
    - In patients with contraindications or intolerance/toxicity to MTX, **leflunomide** (**LEF**, 20 mg/day pos) should be administered next.
    - In patients with contraindications or intolerance/toxicity both to MTX and leflunomide, **sulfasalazine** (**SSZ**, up to 3 gm/day pos) or **hydroxychloroquine** (**HCQ**, 400 mg/day) are the next therapeutic options.
  2. During treatment initiation or disease flares, **glucocorticoids** (prednisolone or its equivalent at a dose of ≤7.5 mg/day) may be added for a short period of time with rapid dose tapering (up to 6 months).
  3. In patients with contraindications or intolerance/toxicity in the above csDMARDs, **monotherapy** with a biologic (bDMARD), or its approved biosimilar) or a targeted synthetic (ts)DMARD) is given:
    - **Biologic DMARDs (bDMARDs)**
      **Anti-Tumour Necrosis Factor - anti-TNFs** (in alphabetic order)
      Adalimumab
      Certolizumab Pegol
      Etanercept
      Golimumab
      Infliximab
      **or**
      **Non-anti-TNFs**
      Abatacept
      IL-6 inhibitors (Tocilizumab or *Sarilumab-EMA approved*)
      IL-1 inhibitors (Anakinra)
      **or**
      **EMA-approved biosimilars**
    - **Janus Kinase (JAK) inhibitor**
      Tofacitinib
      Baricitinib *(EMA approved)*
      Upadacitinib (EMA approved)
      **or**
    - **Rituximab:** Only in patients with history of:
      - Lymphoproliferative diseases or
      - Demyelinating diseases or
      - Solid organ neoplasias
*Step 2*
  1. In patients who fail **csDMARD monotherapy** and in the:
    - **Absence of adverse prognostic factors** (RF and anti-CCP: - and DAS28: 3.2-5.1 and absence of joint erosions),
      - Switching to **or**
      - Addition of a 2^nd^ csDMARD (MTX, LEF, SSZ, HCQ) is recommended
    - **Presence of** ≥**1 adverse prognostic factors** (*Supplementary Table 3*)
      (RF or anti-CCP: +, DAS28 > 5.1, joint erosions), a bDMARD (or its **approved** biosimilar) or targeted synthetic(ts)DMARD is added:
    - **Biologic DMARDs (bDMARDs)**
      **Anti-Tumor Necrosis Factor - anti-TNFs** (in alphabetic order)
      Adalimumab
      Certolizumab Pegol
      Etanercept
      Golimumab
      Infliximab
      **or**
      **Non-anti-TNFs**
      Abatacept
      IL-6 inhibitors (Tocilizumab or *Sarilumab-EMA approved*)
      IL-1 inhibitors (Anakinra)
      **or**
      **EMA-approved biosimilars**
    - **JAK Inhibitor**
      Tofacitinib
      Baricitinib *(EMA approved)*
      Upadacitinib (EMA approved)
      **or**
      **Rituximab:** Only in patients with history of:
      - Lymphoproliferative diseases or
      - Demyelinating diseases or
      - Solid organ neoplasias
Step 3
  1. In patients **who had failed ≥2 or combination of csDMARDs**, a bDMARD (or its **approved** biosimilar) or tsDMARD is added:
    - **Biologic DMARDs (bDMARDs)**
      **Anti-Tumor Necrosis Factor - anti-TNFs** (in alphabetic order)
      Adalimumab
      Certolizumab Pegol
      Etanercept
      Golimumab
      Infliximab
      **or**
      **Non-anti-TNFs**
      Abatacept
      IL-6 inhibitors (Tocilizumab or *Sarilumab-EMA approved*)
      IL-1 inhibitors (Anakinra)
      **or**
      **Their EMA-approved biosimilars**
    - **1^st^ JAK Inhibitor**
      Tofacitinib
      Baricitinib *(EMA approved)*
      Upadacitinib (EMA approved)
      **or**
      **Rituximab:** Only in patients with history of:
      - Lymphoproliferative diseases or
      - Demyelinating diseases or
      - Solid organ neoplasias
  2. In patients who had failed their **1^st^ bDMARD**, a **2^nd^ bDMARD** (or its **approved biosimilar**) or a **tsDMARD** can be added:
    - **2^nd^ bDMARD**:
      **Anti-Tumor Necrosis Factor - anti-TNFs** (in alphabetic order)
        Adalimumab
        Certolizumab Pegol
        Etanercept
        Golimumab
        Infliximab
        **or**
      **Non-anti-TNFs**
        Abatacept
        IL-6 inhibitors (Tocilizumab or *Sarilumab-EMA approved*)
        IL-1 inhibitors (Anakinra)
        **or**
      **EMA-approved biosimilars**
      **1^st^ JAK inhibitor**
        Tofacitinib
        Baricitinib *(EMA approved)*
        Upadacitinib (EMA approved)
  3. In patients who had failed the 1^st^ **JAK Inhibitor**, a 2^nd^ **bDMARD** (or its **approved biosimilar**) or a **2^nd^ tsDMARD,** can be added:
    - **Anti-TNFs** (in alphabetical order)
      Adalimumab
      Certolizumab Pegol
      Etanercept
      Golimumab
      Infliximab
      **or**
      **Non-anti-TNF**
      Abatacept
      IL-6 Inhibitor (Tocilizumab or Sarilumab)
      Anakinra
      Rituximab (after failure of anti-TNF)
      **or**
      **EMA-approved biosimilars**
    - **2^nd^ JAK inhibitor**
      Tofacitinib
      Baricitinib *(EMA approved)*
      Upadacitinib (EMA approved)

### Special Considerations

1. The dose of MTX should be gradually increased up to 20–25 mg/week to achieve the therapeutic target. At doses greater than 15 mg/week, subcutaneous administration of the drug is preferred.
2. In patients with contraindications, intolerance or toxicity to csDMARDs, administration as monotherapy of biological agents is indicated (anti-TNFs: Adalimumab, Certolizumab pegol, Etanercept, anti-IL6: Tocilizumab, *Sarilumab*) or their EMA-approved biosimilars and JAK inhibitors (Tofacitinib, *Baricitinib, Upadacitinib*). More efficacy data regarding monotherapies are available for IL-6 and JAK inhibitors.
3. Among bDMARDs, Anakinra appears to have limited efficacy compared to other bDMARDs (anti-TNFs and non-anti-TNFs).
4. In patients who fail the bio-original DMARDs, changing to their biosimilar is not recommended (or vice versa).
5. In patients with **sustained complete remission** of the disease (as defined by the ACR/EULAR criteria for remission,^6^ *Table 4*) who are being treated with:
  - **csDMARD monotherapy:**
    The following may be attempted:
    - a gradual csDMARD dose reduction,
    - and, only in exceptional cases, its discontinuation
  - **Combination of a csDMARD and a bDMARD**
    The following may be attempted:
    - a gradual dose reduction or an increase in the administration interval of the bDMARD, or
    - a gradual csDMARD dose reduction
  - **Monotherapy with a bDMARD**
    The following may be attempted:
    - a gradual dose reduction or increase in the administration interval of bDMARD
6. There are not adequate data so far to support the discontinuation of bDMARDs in patients with RA at remission.
7. The recommended doses of the different DMARDs are shown in *Supplementary Tables 4–6*.

## CONCLUSIONS

These Guidelines propose a 3-step approach to the treatment of RA targeting low disease activity (DAS28-ESR < 3.2) and always take into consideration the presence or absence of adverse prognostic factors, the presence of comorbidities, and the development of side effects during therapy. Therapeutic decisions should be the result of a shared decision process between the rheumatologist and the well-informed patient.

## Figures and Tables

**Supplementary Table 1:** 

**DAS28-ESR**
The **DAS28-ESR score (0–9.4)** is an established index of disease activity of rheumatoid arthritis. It is calculated by entering the following parameters: Number of tender joints (**TJC,** tender joint count, 0–28 ) Number of swollen joints (**SJC**, swollen joint count, 0–28 ) Erythrocyte Sedimentation Rate (**ESR**, 0–100 mm/h ) Subjective assessment of disease activity by the patient (0–100) The 28 joints assessed for swelling and tenderness are shown in the next graph (grey circles): 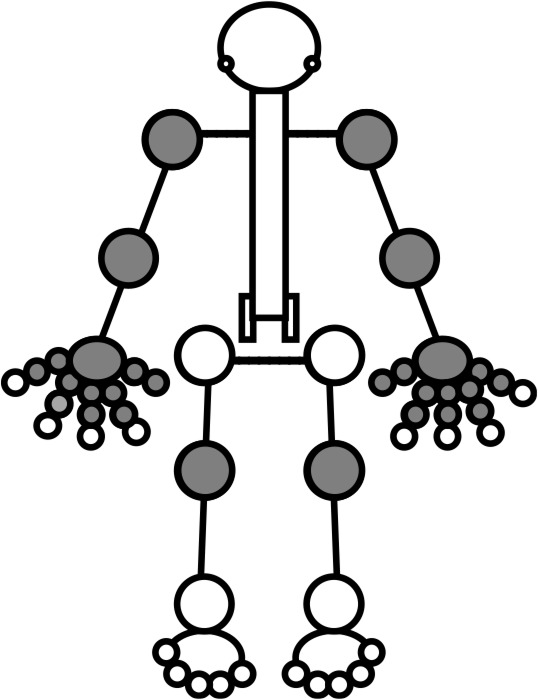 A DAS-calculator is available at the following website: https://www.das-score.nl/das28/DAScalculators/dasculators.html

**Supplementary Table 2:** 

**Disease activity categories according to DAS28-ESR score**
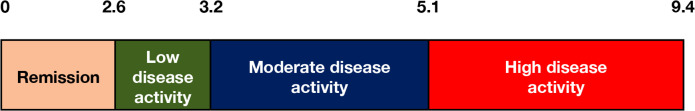

**Supplementary Table 3:** 

**Adverse prognostic factors**
Rheumatoid factor (RF) or anti-CCP + Joint erosions of hands or feet (by plain X-rays) High disease activity score (DAS28-ESR > 5.1)

**Supplementary Table 4:** 

**ACR/EULAR definitions of remission in rheumatoid arthritis**
**Definition 1**
Number of tender joints - TJC **≤1** (0–28) **and** Number of swollen joints - SJC **≤1** (0–28) C-reactive protein (CRP) **≤1 mg/dL and** Patient global assessment **≤1** (scale 0–10 cm) **or** **Simplified Disease Activity Index Activity Index (SDAI) ≤ 3.3** **SDAI (Simplified Disease Activity Index) =** Number of tender joints (0–28) + number of swollen joints (0–28) + CRP (mg/dL) + patient global assessment (scale 0–10 cm) + physician global assessment (scale 0–10 cm)
**Definition 2** Number of tender joints - TJC **≤1** (0–28) **and** Number of swollen joints - SJC **≤1** (0–28) **and** Patient global assessment **≤1** (scale 0–10 cm) **or** **Clinical Disease Activity Index CDAI ≤ 2.8** **CDAI (Clinical Disease Activity Index) =** Number of tender joints (0–28) number of swollen joints (0–28) + patient global assessment (scale 0–10 cm) + physician global assessment (scale 0–10 cm)

**Supplementary Table 5:** 

	**Recommended doses of bDMARDs**
**- Abatacept (ABA)**	< 60 Kg: 500 mg, ≥60-≤100 Kg: 750 mg, > 100 Kg: 1000 mg intravenously (IV) every 4 weeks or 125 mg subcutaneously (SC) every week
**- Adalimumab (ADA)**	40 mg SC every 2 weeks
**- Anakinra (ANA)**	100 mg SC daily
**- Certolizumab Pegol (CZP)**	400 mg SC on weeks 0, 2 καı 4 (loading) and then 200 mg SC every 2 weeks, or upon good clinical response, may be administered at a dose of 400 mg every 4 weeks
**- Etanercept (ETN)**	50 mg SC every week
**- Golimumab (GOL)**	Bodyweight (BW) < 100 kg: 50 mg SC every month BW > 100 kg: Patients who do not achieve an adequate clinical response after 3 or 4 doses with 50 mg SC every month may increase the dose to 100 mg once a month.
**- Infliximab (INFL)**	3 mg/kg IV on weeks 0, 2 καı 6 (loading) and then every 8 weeks. In patients with inadequate response, the dose can be increased up to 7.5 mg/kg every 8 weeks or the interval between doses can be decreased to 3 mg/kg every 4 weeks
- Sarilumab (SAR)^*^	200 mg SC every two weeks or 150 mg SC every two weeks in cases of thrombocytopenia, leukopenia or increase in liver enzymes
**- Tocilizumab (TCZ)**	8 mg/Kg (up to 800 mg) IV every 4 weeks or 162 mg SC every week
**- Rituximab (RTX)**	1000 mg IV on days 0 and 15 for the 1^st^ cycle Repeat cycles every 6 months

The EMA- approved biosimilars are administered at the same dose as the original bDMARDs.

* ***EMA-approved***

**Supplementary Table 6:** 

	**Recommended doses of JAK inhibitors**
**- Tofacitinib (TOFA)**	5 mg pos twice daily or

	5 mg pos once daily, in patients: - with severe renal impairment (creatinine clearance-CrCl: <30 ml/min - with moderate hepatic impairment (Child-Pugh class B) - treated with cytochrome P450 inhibitors (eg, ketoconazole, fluconazole)
- Baricitinib (BAR)^*^	4 mg pos once daily or 2 mg pos once daily, in patients: - older than 75 years - with CrCl: 30–60 ml/min - during dose tapering of patients in remission

- Upadacitinib (UPA)^*^	15 mg pos once daily

* EMA-approved
